# Supplementary material for: Hybrid Models and Biological Model Reduction with PyDSTool
Source: PLoS Comput Biol. 2012 Aug 9;8(8):e1002628. doi: 10.1371/journal.pcbi.1002628 (PMC3415397; doi:10.1371/journal.pcbi.1002628)
Supplement: Text S4 — Complete source code for the PyDSTool package (version 0.88.120504). Includes API documentation and help files linking to web pages. This file is identical to the current public release on Sourceforge.net. (ZIP) [file pcbi.1002628.s004.zip › PyDSTool/html/PyDSTool.parseUtils.parserObject-class.html]

xml version="1.0" encoding="ascii"?


PyDSTool.parseUtils.parserObject


| Home | Trees | Indices | Help | PyDSTool |
| --- | --- | --- | --- | --- |

Package PyDSTool ::
Module parseUtils ::
Class parserObject

[hide private]

[frames] | no frames]

# Class parserObject

source code

```
object --+
         |
        parserObject
```

---

Alphanumeric symbol (pseudo-)parser for mathematical expressions.

An AST is not properly implemented -- rather, we tokenize, identify
free symbols, and apply a small number of syntactic rule checks. The
target language parser is relied upon for full syntax checking.


|  |  |
| --- | --- |
| Instance Methods | [hide private] |

|  |  |
| --- | --- |
| \_\_init\_\_(self, specStr, includeProtected=True, treatMultiRefs=False, ignoreTokens=`[``]`, preserveSpace=False)  x.\_\_init\_\_(...) initializes x; see x.\_\_class\_\_.\_\_doc\_\_ for signature | source code |

|  |  |
| --- | --- |
| isCompound(self, ops=`[``'``+``'``,` `'``-``'``,` `'``*``'``,` `'``/``'``]`)  Function to verify whether an expression is 'compound', in the sense that it has an operator at the root of its syntax parse tree (i.e. | source code |

|  |  |
| --- | --- |
| \_\_call\_\_(self, specialtoks=None, symbolMap=None, includeProtected=True) | source code |

|  |  |
| --- | --- |
| find(self, token)  Find all occurrences of the given token in the expression, returning a list of indices (empty if not present). | source code |

|  |  |
| --- | --- |
| parse(self, specialtoks, symbolMap=None, includeProtected=True, reset=False) | source code |

**Inherited from `object`**:
`__delattr__`,
`__getattribute__`,
`__hash__`,
`__new__`,
`__reduce__`,
`__reduce_ex__`,
`__repr__`,
`__setattr__`,
`__str__`


|  |  |
| --- | --- |
| Properties | [hide private] |

**Inherited from `object`**:
`__class__`


|  |  |
| --- | --- |
| Method Details | [hide private] |

|  |  |
| --- | --- |
| \_\_init\_\_(self, specStr, includeProtected=True, treatMultiRefs=False, ignoreTokens=`[``]`, preserveSpace=False)  *(Constructor)* | source code |

x.\_\_init\_\_(...) initializes x; see x.\_\_class\_\_.\_\_doc\_\_ for
signature

Overrides: object.\_\_init\_\_: *(inherited documentation)*

|  |  |
| --- | --- |
| isCompound(self, ops=`[``'``+``'``,` `'``-``'``,` `'``*``'``,` `'``/``'``]`) | source code |

Function to verify whether an expression is 'compound', in the sense
that it has an operator at the root of its syntax parse tree (i.e. not
inside braces).

  


| Home | Trees | Indices | Help | PyDSTool |
| --- | --- | --- | --- | --- |

|  |  |
| --- | --- |
| Generated by Epydoc 3.0.1 on Fri May 4 15:24:10 2012 | http://epydoc.sourceforge.net |
